# The wHole Story About Fenestrations in LSEC

**DOI:** 10.3389/fphys.2021.735573

**Published:** 2021-09-13

**Authors:** Karolina Szafranska, Larissa D. Kruse, Christopher Florian Holte, Peter McCourt, Bartlomiej Zapotoczny

**Affiliations:** ^1^Vascular Biology Research Group, Department of Medical Biology, University of Tromsø – The Arctic University of Norway, Tromsø, Norway; ^2^Department of Biophysical Microstructures, Institute of Nuclear Physics, Polish Academy of Sciences, Kraków, Poland

**Keywords:** fenestration, fenestra, nanopores, LSEC, liver sinusoidal endothelial cells, porosity, liver disease, drug response

## Abstract

The porosity of liver sinusoidal endothelial cells (LSEC) ensures bidirectional passive transport of lipoproteins, drugs and solutes between the liver capillaries and the liver parenchyma. This porosity is realized via fenestrations – transcellular pores with diameters in the range of 50–300 nm – typically grouped together in sieve plates. Aging and several liver disorders severely reduce LSEC porosity, decreasing their filtration properties. Over the years, a variety of drugs, stimulants, and toxins have been investigated in the context of altered diameter or frequency of fenestrations. In fact, any change in the porosity, connected with the change in number and/or size of fenestrations is reflected in the overall liver-vascular system crosstalk. Recently, several commonly used medicines have been proposed to have a beneficial effect on LSEC re-fenestration in aging. These findings may be important for the aging populations of the world. In this review we collate the literature on medicines, recreational drugs, hormones and laboratory tools (including toxins) where the effect LSEC morphology was quantitatively analyzed. Moreover, different experimental models of liver pathology are discussed in the context of fenestrations. The second part of this review covers the cellular mechanisms of action to enable physicians and researchers to predict the effect of newly developed drugs on LSEC porosity. To achieve this, we discuss four existing hypotheses of regulation of fenestrations. Finally, we provide a summary of the cellular mechanisms which are demonstrated to tune the porosity of LSEC.

## Introduction

**FIGURE 1:**
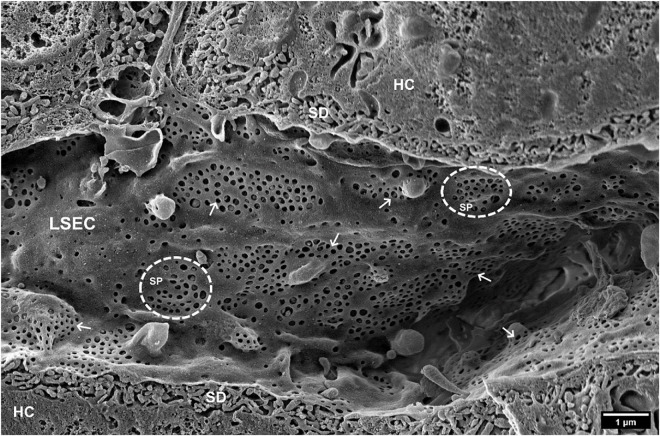
SEM image of hepatic sinusoids of a C57BL6 mouse, approximately 4 months old. Liver Sinusoidal Endothelial Cells (LSECs) are covered in multiple fenestrations (arrows) arranged into sieve plates (SP, dotted line circles) distributed over the whole sinusoid. SD, space of Disse; HC, hepatocytes. (Courtesy of Karen K. Sørensen, UiT, Tromsø, Norway).

Within the human body, the main blood-organ barrier is made up of a single layer of thin endothelial cells. In the liver, the microcirculation has a unique morphology that facilitates bi-directional exchange of substrates between hepatocytes and blood in the liver sinusoids (Cogger and Le Couteur, 2009; Fraser et al., 2012). Liver sinusoidal endothelial cells (LSEC) are very thin and perforated with transcellular pores (50–300 nm in diameter) that are also termed as fenestrae or fenestrations (Figure 1). These structures were first correctly identified as such with transmission electron microscopy (TEM) by Yamagishi (1959) and described in detail by Wisse (1970). Between 2 and 20% of the LSEC surface is covered by fenestrations which are either scattered individually across the surface or clustered into groups called sieve plates. As there are no diaphragms or underlying basement membrane, fenestrations make LSEC a highly efficient ultrafiltration system. LSEC thus retain blood cells inside the vessel lumen, whereas small molecules, such as drugs, proteins, lipoproteins, and small viruses can pass this endothelial barrier via fenestrations to reach the surrounding hepatocytes, and vice versa (Fraser et al., 1995a). Fenestrations are therefore a vital structure in liver physiology, providing the primary communication conduit between the liver and the rest of the body, via the circulation. LSEC fenestrations, and the effects of various agents upon them, have been studied extensively with electron microscopy. During the last decade new techniques have been developed and became available to investigate fenestrations in cultured LSEC. Super-resolution optical microscopy provided first detailed information about the composition of fenestration (Cogger et al., 2010, 2013; Mönkemöller et al., 2015; Zapotoczny et al., 2019a) while atomic force microscopy (AFM) provided first information about the dynamics of fenestrations *in vitro* (Zapotoczny et al., 2019b, 2020). Such tools will accelerate the development of therapies that can reverse the loss of fenestrations seen in aging and liver fibrosis (DeLeve, 2015; Hunt et al., 2019).

**FIGURE 2:**
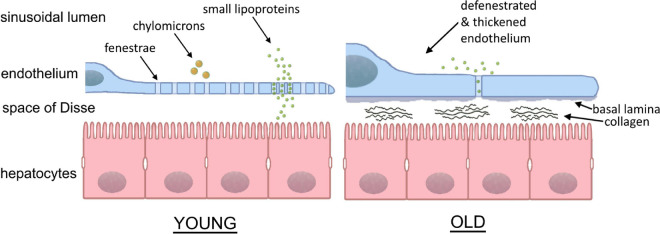
Sinusoidal lumen in young and old liver. With age, the fenestrated morphology of the sinusoids is lost in the process of “pseudocapillarization.” Additionally, the endothelium thickens and collagen deposits can be found within the space of Disse. The result is the inhibition of transfer between the blood and hepatocytes. (Courtesy of Eike Struck, UiT, Tromsø, Norway and David Le Couteur, ANZAC Research Institute, Sydney, Australia).

Fenestration loss during aging manifests as changes in the liver microcirculation, in particular within LSEC, which is a likely cause of dyslipidemia (Le Couteur et al., 2002) and insulin resistance in old age (Mohamad et al., 2016). At the morphological level, LSEC in old age have markedly reduced porosity (percent of the cell surface area covered in fenestrations) by about 50% – in other words, old LSEC become “defenestrated” (Figure 2). This defenestration results in hampered bi-directional traffic of substrates between the blood and the hepatocytes. Biomolecules such as lipoproteins, or hormones, or drugs (such as statins or insulin) pass less easily through aged LSEC to reach the hepatocytes to be processed and/or exert their effects. For example, older rats showed a significant reduction in the hepatic volume of insulin distribution (Mohamad et al., 2016), showing that fenestrations facilitate insulin transfer to hepatocytes. Another example is the transfer of lipoproteins across LSEC, which was almost totally abolished in livers from old animals, providing a novel mechanism for age-related dyslipidemia and postprandial hyperlipidemia (Hilmer et al., 2005) and is now accepted as a significant factor in age-related hyperlipidemia (Liu et al., 2015). The same applies in the reverse direction across LSEC – biomolecules produced by the hepatocytes need to pass through fenestrations for release into the plasma, and defenestration hinders this process. Age-related LSEC defenestration is also accompanied by altered expression of many vascular proteins including von Willebrand factor, ICAM-1, laminin, caveolin-1 and various collagens (Le Couteur et al., 2008). However, these changes occur without any age-related pathology of hepatocytes or activation of stellate cells (Warren et al., 2011). The sum of all these processes results in a state whereby liver sinusoidal vessels become more like continuous capillaries, but without the other manifestations seen in diseased livers during “capillarization.” Age-related defenestration is therefore also termed “pseudocapillarization.” Cellular senescence is one hallmark of aging (Robbins et al., 2021), and (Grosse et al., 2020) proposed that LSEC become senescent at 10–12 months of age in mice, as evidenced by the increased expression of the senescence marker p16. Senolytic drugs (which selectively kill senescent cells) have been proposed as a potential therapy to alleviate the effects of senescent cell mediated aging and disease (Robbins et al., 2021). However, p16^high^ LSEC are essential for mouse healthspan, as ablation of these cells results in disruption of the hepatic sinusoid and liver fibrosis (Grosse et al., 2020).

Defenestration of LSEC also occurs during chronic liver disease, liver fibrosis and consequently cirrhosis, which are an increasing worldwide problem, and are becoming a major cause of morbidity and death (Asrani et al., 2019). Currently, there is no therapy that can alleviate fibrosis progression or reverse fibrosis (Higashi et al., 2017). Fibrosis is characterized by excessive extracellular matrix production from activated stellate cells. In addition to LSEC defenestration, during chronic liver disease, a basement membrane develops in the Space of Disse, leading to the process of capillarization, and thereby further reducing the free passage of substrates to and from the hepatocytes (Poisson et al., 2017). Defenestration of LSEC occurs earlier than the formation of fibrous septa in liver diseases such as alcoholic liver injury and non-alcoholic fatty liver disease (Horn et al., 1987) which could indicate that LSEC can play an important role during the early stages of fibrosis. Restoration of differentiation to LSEC led to quiescence of hepatic stellate cells and regression of fibrosis in thioacetamide challenged rats (Xie et al., 2012b) potentially suggesting that therapies that revert LSEC from a diseased/defenestrated state to a normal state may also be of benefit for treatment of liver fibrosis (DeLeve, 2015).

As mentioned above, defenestration of the liver sinusoidal endothelium impairs the hepatic clearance of pharmacological agents (Mitchell et al., 2011). As for lipoproteins and insulin, fenestrations are conduits for pharmaceuticals, from the plasma to the hepatocytes. Reduction in LSEC porosity thus reduces the passage of drugs to the cells where they are processed and metabolized. This can result in elevated and potentially toxic concentrations of drugs in the elderly (and patients with liver disease), when administering drug doses appropriate for healthy young people. In addition, polypharmacy is becoming a major issue in the aging population, with over 42% of people over 65 years of age were reported being administrated five or more different medications per day (Midão et al., 2018). The majority of these medications need to cross the liver sinusoidal endothelium to be detoxified, and it is possible that some of the polypharmacy “cocktails” are detrimental for LSEC porosity. Another serious consequence of reduced porosity is that statins are less able to reach the hepatocytes and inhibit cholesterol production. Increased statin doses are then required to achieve therapeutic effects, sometimes resulting in side effects such as muscle pain and rhabdomyolysis, resulting in medication non-compliance in patients.

Given the vital role of LSEC fenestrations (and the bi-directional flow of substrates through them) in physiology and homeostasis, a better understanding of how these structures are regulated will enable us to design novel therapeutic approaches targeting biological changes of aging and liver diseases.

It needs to be highlighted, however, that many reports in the literature “suffer” from developing experimental methodologies. Errors during liver perfusion, cell isolation methodologies and sample preparations may lead to altered cell phenotypes. Also, it should be noted that studies from pre-super-resolution era where light microscopy was the only technique used for quantification of fenestrations may be imprecise. As reported, fenestrations in LSEC are in the range of 50–300 nm, gathered in sieve plates of several to tens of pores, with limited number of gaps (DeLeve and Maretti-Mira, 2017). These can be visualized only using non-diffraction limited methods such as electron microscopy, optical nanoscopy, or atomic force microscopy. The distribution of fenestration diameter in this range was presented for both LSEC in tissue (*in vivo*) and for isolated cells (*in vitro*). *In vivo* data are limited to fixed and dried material, while data for isolated LSEC covers fixed and dried, wet-fixed, and live cells. Recently, we summarized that the differences in mean fenestration diameter for fixed and dried, wet-fixed and live LSECs *in vitro* can be up to 30% (Supplementary Table 1 in Zapotoczny et al., 2019b). The differences between *in vivo* and *in vitro* data can be even larger *ibid*., (Wisse et al., 2010). The comparison between the groups in a single report provides information about the alterations as the same microscopy method is applied. The methodological details enabling avoiding errors in imaging and data analysis were described: SEM (Wisse et al., 2010; Cogger et al., 2015; Szafranska et al., 2021), AFM (Zapotoczny et al., 2017a, 2020; Szafranska et al., 2021), SIM (Kong et al., 2021; Szafranska et al., 2021). Moreover, the comparative measurements using different microscopies were reported in the past showing good correlation between the methods. However, the comparative analysis of newly developed techniques applied recently for LSECs, such as SIM, STED, and AFM, is lacking. Each method has its advantages and limitations. To enable easy tracking of the model (*in vivo/in vitro* and microscopy technique) we provide the relevant information in the presented tables.

The purpose of this review is to: (i) provide a medical and cell biology “tool-kit,” for researchers and clinicians to design potential LSEC refenestration strategies and (ii) summarize the existing knowledge around fenestration biology which can help to find new ways to reveal how fenestrations actually work. The first part of this review fucuses on the reported influence of drugs on LSEC fenestration number and porosity, while the second part gives a deeper knowledge about fenestration biology and mechanisms behind structure, formation and maintenance of fenestration. This review does not cover a number of other aspects of LSEC biology, but these can be found in in the following excellent reviews about LSEC in: diseases (Gracia-Sancho et al., 2021; Wang and Peng, 2021), hepatic fibrosis (DeLeve, 2015), mechanotransduction (Shu et al., 2021), inflammation and cancer (Wilkinson et al., 2020; Yang and Zhang, 2021), receptor expression (Pandey et al., 2020), immunological functions (Shetty et al., 2018), aging (Hunt et al., 2019), scavenging (Sørensen et al., 2012), and overall biology of LSECs (Sørensen et al., 2015).

## LSEC and Drug Interactions

### Recreational and Medicinal Drugs, and Their Effects on LSEC Porosity

The human race already uses an extensive array of drugs for medical and recreational purposes. The majority of these compounds are safe, or at least relatively safe for normal human consumption if used appropriately. Reported negative side-effects of these drugs are typically well-documented at the systemic or organ level, but little is known about their direct effects on LSEC fenestration status. Additionally, some drugs with other intended targets may actually have positive side effects on LSEC fenestration, leading to increased LSEC porosity and improving bi-directional exchange of solutes between hepatocytes and plasma. This concept was first tested by Hunt et al. (2019, 2020) who found that a number of drugs for intended use for the treatment of high blood pressure, erectile dysfunction and diabetes improved LSEC porosity in young and old mice. Table 1 lists the effects of some recreational and medicinal drugs on LSEC fenestrations.

**TABLE 1:** Influence of medicinal drugs on LSEC fenestrations.

	Fenestration diameter	Porosity	Fenestration frequency	References	Methods
**Recreational drugs**					
Ethanol	+/-	-	-	Van Der Smissen et al., 1986; Braet et al., 1995a	SEM, TEM, *in vitro*
				Mak and Lieber, 1984; Charles et al., 1986; de Zanger et al., 1997	SEM, *in vivo*
				Tanikawa et al., 1991; McCuskey et al., 1993	TEM, *in vivo*
				Horn et al., 1987; Takashimizu et al., 1999	SEM, *in vivo*
				Braet et al., 1996c	SEM, AFM, *in vitro*
				Braet et al., 1994	SEM, *in vitro*
Ethanol +cocaine	nd	-	-	McCuskey et al., 1993	TEM, *in vivo*
Cocaine	nd	nd	nd	McCuskey et al., 1993	TEM, *in vivo*
2,5-Dimethoxy-4-iodoamphetamine (DOI)	+	+/-	+/-	Furrer et al., 2011; Cogger et al., 2014	SEM, *in vivo*
				Hunt et al., 2019	SEM *in vitro*
Nicotine	-	- -	-	Fraser et al., 1988	SEM, *in vivo*
**Prescription drugs**					
Acetaminophen/paracetamol +ethanol	G	nd	nd	McCuskey et al., 2004	SEM, TEM, *in vivo*, *in vitro*
Acetaminophen/paracetamol	G	-	-	Ito et al., 2006b	SEM, *in vivo*
				Walker et al., 1983	SEM, TEM, *in vivo*
				McCuskey et al., 2004; McCuskey, 2006	SEM, TEM, *in vivo*, *in vitro*
Amlodipine	-	+	+	Hunt et al., 2019	SEM, *in vitro*
Bosentan	0	+	+	Hunt et al., 2019	SEM, *in vitro*
Colchicine	nd	nd	0	Braet et al., 1996b	TEM, *in vitro*
Disulfiram	-	nd	+	Bernier et al., 2020	SEM, *in vivo*
Metformin	0	+	+	Hunt et al., 2020	SEM, *in vitro*, *in vivo*
				Alfaras et al., 2017	SEM, *in vivo*
Nicotinamide mononucleotide (NMN)	0	+	+	Hunt et al., 2019	SEM, *in vitro*
				Mao et al., 2019	dSTORM, *in vitro*
Cholesterol	0	0	0	Fraser et al., 1988, 1989	SEM, *in vivo*
Cholesterol +nicotine	-	-	-	Fraser et al., 1988	SEM, *in vivo*
Pantethine + cholesterol	+	+	+	Fraser et al., 1989	SEM, *in vivo*
Prostaglandin E1	+			Oda et al., 1997	SEM, TEM, *in vitro*
Sildenafil	0/+	++	+	Hunt et al., 2019	SEM, *in vitro*
				Mao et al., 2019	dSTORM, *in vitro*
Simvastatin	+	+	+	Hide et al., 2020	SEM, TEM, *in vivo*, SEM, *in vitro*
				Venkatraman and Tucker-Kellogg, 2013; Hunt et al., 2019	SEM, *in vitro*
Taxol	nd	nd	0	Braet et al., 1996b	TEM, *in vitro*
TNF-related apoptosis-inducing ligand (TRAIL)	+/0	+/0	+/0	Hunt et al., 2019	SEM, *in vitro*

*“0,” no change; G, gaps; increase: “+,” <50%; “++,” 50–100%; “+++,” >100%; decrease: “-,” <50%; “- -,” >50%; “- - -,” defenestration; “nd,” no data.*

#### Recreational Drugs

The effects of recreational drugs on LSEC porosity have not been studied extensively (Table 1). The few studies performed showed that the recreational drugs nicotine, ethanol, and cocaine reduce LSEC porosity (Fraser et al., 1988; McCuskey et al., 1993), while the psychedelic drug 2,5-Dimethoxy-4-iodoamphetamine (DOI) increases porosity in LSEC in young and old rodents (Cogger et al., 2014; Hunt et al., 2019). The effects on LSEC porosity of other recreational/non-medicinal drugs such as opioids, amphetamines, cannabis, and xanthines (such as caffeine and theobromine) have, to the best of our knowledge, not been studied. This would be an area of great interest, given the extensive use of all of these among the general population. This is exemplified by opioid use (which is also for medicinal purposes) leading to the current “opioid epidemic” in the US arising from the use of prescription oxycodone. Below is a summary of the reported interactions of ethanol, cocaine, DOI, and nicotine with LSEC.

**Ethanol** Given the wide use and general acceptance of alcohol, and the suggested health benefits from moderate consumption, it was discussed in the LSEC field whether moderate amounts of alcohol could improve LSEC porosity and thereby lipoprotein clearance. Of the studies (*in vitro* and *in vivo*) investigating the effects of ethanol on LSEC, the majority were performed in rats, but mice, baboons and human LSEC were studied as well, with electron and atomic force microscopy methods used as readout. Several studies reported that the fenestration number was reduced, while the average fenestration diameter was increased – this pattern was consistent in all the *in vitro* studies (Mak and Lieber, 1984; Charles et al., 1986; Van Der Smissen et al., 1986; Horn et al., 1987; Tanikawa et al., 1991; McCuskey et al., 1993; Braet et al., 1994, 1995a, 1996c; de Zanger et al., 1997) and with reduced porosity reported in one study (Takashimizu et al., 1999). Takashimizu et al. (1999) described reduction in fenestration diameter in rat during *in vivo* continuous administration of ethanol into the portal vein, and pre-treatment with **BQ123** [an endothelin (ET) receptor antagonist, see Table 2] reduced the effect of ethanol. One *in vivo* study reported no changes in in the liver sinusoids in mice after 9 weeks of ethanol feeding (McCuskey et al., 1993) but ethanol in combination with cocaine caused the sinusoids to become thickened and defenestrated. In other *in vivo* chronic ethanol challenge studies (ethanol given to rats in food, or human studies where biopsies were used), one rat study yielded results consistent with the *in vitro* findings (reduced fenestration number, increased diameter, reduced porosity) (Tanikawa et al., 1991) while the other study reported reduced fenestration diameter and number – this was the only study to find that the diameter became smaller after ethanol challenge (Takashimizu et al., 1999). In the human biopsy study, similar results were obtained - chronic alcohol consumption (defined as > 60 g alcohol intake every day for more than 3 years) resulted in fewer fenestrations, diameters of between 50–300 nm and a “visible difference” for porosity between the two groups. A study in baboons showed that the duration of alcohol consumption does not seem to have any impact on fenestrations (diameter in second group (4–24 months alcohol consumption vs. 61–112 months) was larger than control but smaller than first group) (Mak and Lieber, 1984). In summary, ethanol at any dose does not appear to improve LSEC porosity but rather has the opposite effect.

**TABLE 2:** Influence of hormones and other agents acting on LSEC fenestrations.

	Fenestration diameter	Porosity	Fenestration frequency	References	Methods
**Vasoactive stimuli**					
**Vasodilators**					
Acetylcholine	+	nd	nd	Tsukada et al., 1986; Oda et al., 1990	SEM, *in vivo*, *in vitro*
Bethanechol	+	nd	nd	Oda et al., 1990	SEM, *in vivo*
Isoproterenol	+	nd	nd	Oda et al., 1990	SEM, *in vivo*, *in vitro*
Vasoactive intestinal peptide (VIP)	+	nd	nd	Oda et al., 1990	SEM, *in vivo*
BQ-123	++	nd	-	Watanabe et al., 2007	SEM, TEM, *in vivo*
**Vasoconstrictors**					
Endothelin (ET)	-	-	nd	Oda et al., 1997; Kamegaya et al., 2002	SEM, *in vitro*
Neuropeptide Y	-	nd	nd	Oda et al., 1990	SEM, *in vivo*
Norepinephrine/noradrenaline	-	nd	nd	Tsukada et al., 1986; Oda et al., 1990	SEM, *in vivo*, *in vitro*
				Wisse et al., 1980	TEM, SEM, *in vivo*
Serotonin	-	nd	nd	Wisse et al., 1980; Braet et al., 1995a	SEM, TEM, *in vivo*
				Tanikawa et al., 1991	TEM, *in vivo*
				Braet et al., 1996c	SEM, AFM, *in vitro*
				Kalle et al., 1997	AFM, *in vitro*
Pilocarpin	-	nd	nd	Wisse et al., 1980	TEM, SEM, *in vivo*
Adrenaline/epinephrine	-	nd	nd	Wisse et al., 1980	TEM, SEM, *in vivo*
**Signaling/Maintenance**					
Vascular endohelial growth factor (VEGF)	+	+++	++	Funyu et al., 2001; Yokomori et al., 2003	SEM, *in vitro*
				Carpenter et al., 2005	SEM, TEM, *in vivo*
				Xie et al., 2012b	SEM, *in vivo*, *in vitro*
Bone morphogenetic protein (BMP)	Strain specific	Strain specific	Strain specific	Desroches-Castan et al., 2019a,b	(a) SEM, *in vivo*, *in vitro* (b) SEM, *in vitro*
Platelet derived growth factor (PDGF-B) signaling	nd	-	nd	Raines et al., 2011	TEM, *in vivo*
Liver X receptor (LXR)	NA	NA	NA	Xing et al., 2016	SEM, TEM, *in vivo*
Hedgehog (Hh) signaling	nd	-	nd	Xie et al., 2012a	SEM, *in vitro*
Plasmalemma vesicle associated protein (PLVAP)	+/-	+/-	+/-	Herrnberger et al., 2014	SEM, TEM, *in vivo*
				Auvinen et al., 2019	SEM, *in vivo*

*“0,” no change; G, gaps; “nd,” no data; “NA,” not applicable. increase: “+,” <50%; “++,” 50–100%; “+++,” >100%; decrease: “-,” <50%; “- -,” >50%; “- - -,” defenestration.*

**Cocaine** is a widely used recreational drug with vasoconstricting properties (Kim and Park, 2019), often consumed in combination with alcohol. In a study from McCuskey et al. (1993), mice challenged with cocaine alone developed basement membrane deposition in the space of Disse, some hepatocellular necrosis and slightly reduced centrilobular sinusoid blood flow after 5 weeks, worsening up to 9 weeks of challenge. In combination with ethanol these changes were significantly exacerbated, in addition the sinusoidal endothelium was thickened and defenestrated. Interestingly rats were more resistant to these challenges, only developing some of these changes at the end of the 15-week treatment regime. The mechanism(s) by which cocaine and cocaine/ethanol challenge elicit these changes remains to be elucidated, but in any case the combined abuse of these drugs raises particular concerns with regards to liver function.

**Nicotine** is the primary stimulant found in tobacco products and is also a known vasoconstrictor (Benowitz and Burbank, 2016). Rats fed nicotine (dose equivalent to 50–100 cigarettes per day in humans for 6 weeks) had LSEC porosity 40% of that of controls, primarily as a function of reduced average fenestration diameter and not of reduced fenestration number. The nicotine treated animals also had near 50% higher serum cholesterol than controls, probably as a consequence of reduced LSEC porosity and thereby filtration of low-density lipoprotein (LDL) out from the plasma of these animals (Fraser et al., 1988). Nicotine and cholesterol fed animals had similar porosity and diameter to nicotine-fed only animals. Together with results from cholesterol-only fed animals (no visible changes), it suggests that nicotine (but not cholesterol) has an effect on fenestrations (Fraser et al., 1988). Other studies have shown that oral nicotine induces an atherogenic lipoprotein profile (Cluette-Brown et al., 1986) (including increased plasma LDL) and impairs plasma LDL clearance (Hojnacki et al., 1986). The mechanism of action of nicotine in the LSEC context remains to be elucidated but given the continued consumption of nicotine by humans in various forms (e.g., tobacco products, e-cigarettes, and nicotine supplements) this field warrants further study.

**2,5-Dimethoxy-4-iodoamphetamine** (DOI) is a substituted amphetamine but is not a stimulant. It is a potent 5-HT_2__A_ serotonin receptor agonist and is used recreationally as a hallucinogenic drug (Lapoint et al., 2013). DOI induces cutaneous vascular constriction in rabbits and rats, and this is the suggested cause of hyperthermia resulting from serotonin receptor stimulation (Blessing and Seaman, 2003). DOI has reported beneficial effects on survival, liver regeneration and LSEC morphology after partial hepatectomy (Tian et al., 2011). Furrer et al. (2011) showed that *in vivo* DOI challenge increased porosity in old but not young LSEC, and pre-treatment of old mice with DOI prior to partial hepatectomy resulted in LSEC with improved porosity (Furrer et al., 2011). However, the finding that DOI improved porosity in aged LSEC is at odds with the *in vivo* study of Cogger et al. (2014) who found that DOI improved LSEC porosity in young but not old animals. Both studies used SEM of tissue blocks to quantify fenestrations. Further complicating the DOI story, SEM *in vitro* studies by Hunt et al. (2019) on cultured LSEC from young and old mice revealed that DOI challenge increased porosity in old but not young LSEC, and this increase was most likely a function of increase in both fenestration diameter and frequency. LSEC respond to ligands for the 5-HT2 receptor, as they were reported to being inhibited by ketanserin (a selective 5-HT2 receptor antagonist) (Gatmaitan et al., 1996). The role of 5-HT2A and 2B receptors was proposed as being involved in liver regeneration after liver partial hepatectomy (Lesurtel et al., 2006). Similarly, the presence of the 5HT2 receptor was later highlighted (Braet and Wisse, 2002; Braet, 2004). However, newly reported data showed that known 5-HT receptor mRNAs were absent or at very low levels in mouse, rat and human LSEC (Bhandari et al., 2020). It would thus be of interest to resolve the question of DOI mediated effects, the downstream mechanisms, and whether there is/are age-related responses to DOI.

#### Medicinal Drugs

Pharmaceutical treatment and prevention of diseases is constantly evolving, with an increasing number of novel medicines entering the market every year. It was reported that the EU retail pharmaceutical bill was around EUR 190 billion in 2018 (OECD/European Union, 2020). Hepatic clearance and metabolism are the basic routes of removing drugs from the system. With decreased porosity prolonged circulation of drugs increases their side effects. Nitric oxide (NO)-based drug therapy was shown to have beneficial effects on the liver (Maslak et al., 2015) and detailed studies on isolated cells confirm the positive role of NO on fenestrated morphology in LSEC (Xie et al., 2012b). Medicinal drugs with other intended targets may also affect LSEC. A recent comparative study revealed the different drug effects on fenestrations in LSEC in an age-related manner (Hunt et al., 2019). Here we summarize the effects of various medicines where fenestration number and size were reported.

**Amlodipine** is a calcium channel blocker used to treat hypertension by dilating blood vessels to reduce blood pressure. Amlodipine is also reported to increase endothelial NO (Xu et al., 2002; Mason et al., 2014). Hunt et al. (2019) reported that amlodipine increased the porosity in cultured LSEC from both young and old animals and proposed that this increase was more likely mediated by NO production than by calcium transport blockage. This safe and commonly used blood pressure medicine may thus also represent a pharmacological means to counteract age-related defenestration.

**Bosentan** is a competitive antagonist of endothelin -A and -B receptors, and is used to treat moderate pulmonary hypertension, exerting its vasodilative effect via ET-A receptors (Bacon et al., 1996). Endothelin-1 (ET-1) constricts fenestrations pronouncedly and reduces porosity (Kamegaya et al., 2002), and an ET-B receptor antagonist (BQ788) blocked this effect while an ET-A receptor antagonist (BQ485) partially blocked the ET-1 effect (Kamegaya et al., 2002). The ET-A receptor antagonist **BQ123** increased fenestration diameters, but caused major gaps in sinusoidal cells and fusions of fenestrations within sieve plates (Watanabe et al., 2007). Hunt et al. (2019) demonstrated that lower doses of bosentan increased the porosity of LSEC from old mice, while LSEC from younger mice were non-responsive. Bosentan treatment of LSEC did not elicit an increase in NO production in this study.

**Colchicine** is used as a therapy for gout and familial Mediterranean fever. It decreases inflammation but its pharmacotherapeutic mechanism of action is not fully understood – its main mechanism of action is tubulin disruption (Leung et al., 2015). Treatment of cultured rat LSEC with 200 μM colchicine did not affect porosity while causing significant loss of microtubules. Interestingly, the microtubules surrounding sieve plates were still present (Braet et al., 1996b). Together with the effect of taxol, which completely disrupts microtubules and prevents cytochalasin-mediated induction of fenestrations, this would suggest that tubulin architecture may have a crucial role in LSEC porosity. **Taxol** (generic name paclitaxel) is a microtubule-stabilizing drug used for the treatment of ovarian, breast, and lung cancer, as well as Kaposi’s sarcoma (Weaver, 2014). Braet et al. (1996b) challenged cultured rat LSEC with 10 μM taxol and saw no change in porosity but reported an overabundance of microtubules throughout the cytoplasm, and alongside sieve plates. Moreover, treatment with 10 μM taxol not only did not show a significant change in fenestration number but pretreatment with taxol and two hours later with cytochalasin B, inhibits the effect of the latter, i.e., the increase in fenestration number is reduced in comparison to treatment with cytochalasin B only.

**Disulfiram** (commercial name Antabuse) is a FDA approved treatment for chronic alcohol addiction. It is an inhibitor of acetaldehyde dehydrogenase and causes the feeling of a hangover immediately upon alcohol consumption (Suh et al., 2006). It is an inhibitor of the transcription factor NF-KB (Schreck et al., 1992) which contributes to its anti-inflammatory properties. In the experimental setting, the consumption of disulfiram was found to normalize body weight in mice. It was also found to increase the frequency of LSEC fenestrations *in vivo*, while decreasing their average diameter, resulting in no net increase in porosity in mice and rats (Bernier et al., 2020). The mechanism(s) by which disulfiram increases fenestration number remain to be elucidated.

**Metformin** is a first line treatment for type II diabetes for serum glucose reduction (Maruthur et al., 2016). The mechanism by which this drug exerts this effect remains to be elucidated, but its primary target appears to be hepatocyte mitochondria via inhibition of complex I of the respiratory chain. Inhibition of gluconeogenesis (Owen et al., 2000) results in the activation of the energy sensor AMP-activated protein kinase (AMPK) leading to increased beta-oxidation of fatty acids. Alfaras et al. (2017) tested 1% metformin administered every-other-week or 2-weeks-every-month to mice – these strategies being chosen to avoid metformin induced nephrotoxicity. They found numerous health benefits, particularly with the every-other-week regime, and that the every-other-week approach also increased porosity in LSEC in 2-year-old mice. Metformin (50 μM) increased LSEC porosity *in vitro* in both young and old mice by 25 and 50%, respectively (Hunt et al., 2020). This increase was due to increases in fenestration frequency (20 and 50%, respectively) since the fenestration diameter remained unchanged. *In vivo* studies in mice treated with 0.1% metformin in their diet increased LSEC porosity/fenestration frequency in young and old mice and reduced the age-related loss of porosity in older mice by 50% (Hunt et al., 2020). The mechanism of metformin action in LSEC, with regards to fenestration status, remains to be established.

**Nicotinamide mononucleotide** (NMN) is a key nicotinamide adenine dinucleotide (NAD+) intermediate. Long-term administration of NMN is reported to mitigate age-related physiological decline in mice (Mills et al., 2016), while short term *in vitro* treatment reverses endothelial dysfunction (Mateuszuk et al., 2020). NMN increased LSEC porosity in young and old mice, via increased fenestration frequency, while the average fenestration diameter was essentially unchanged (Hunt et al., 2019). NMN challenge had no apparent effects on NOS or cGMP levels in LSEC. Analysis of NMN challenged LSEC using direct stochastical optical reconstruction microscopy (dSTORM) revealed that the F-actin within LSEC was more condensed and that the actin rings delineating fenestrations became more pronounced (Mao et al., 2019). The mode of NMN action in LSEC remains to be elucidated – NAD + associates with sirtuins which play a critical role in multiple cellular functions (Imai and Yoshino, 2013) so the study of the role of sirtuins in fenestration biology is therefore warranted.

**Pantethine** is a derivative of vitamin B5 and has been suggested as a therapy for reducing LDL levels (Rumberger et al., 2011). Fraser et al. (1989) studied the effect of pantethine in cholesterol fed rabbits. The pantethine plus cholesterol fed animals had higher LSEC porosity, fenestration diameter and frequency and lower total cholesterol than the animals fed cholesterol alone. **Cholesterol** feeding had no effect on LSEC porosity. The same result had been found in another study (Fraser et al., 1988). Unfortunately, there was no group fed only pantethine, so it would be interesting to establish if pantethine alone increases LSEC porosity and if this can explain (in part) the reported pantethine-mediated reduction of plasma LDL seen in other studies (Fraser et al., 1989; Rumberger et al., 2011).

**Paracetamol** (also known as acetaminophen or commercially as APAP, Panadol) is one of the most widely used analgesic medicines. Acute overdoses of paracetamol can cause lethal liver damage, due to the toxic metabolite *N*-acetyl-*p*-benzoquinone imine (NAPQI) (Hodgman and Garrard, 2012). The consensus is that, *in vivo*, paracetamol reduces rodent LSEC porosity both via reduction of fenestration diameter and frequency at “clinical” doses (Walker et al., 1983; McCuskey et al., 2004; McCuskey, 2006; Ito et al., 2006b). The *in vitro* effect of paracetamol on LSEC was reported to be dependent on NAPQI induced depletion of glutathione levels. In C3H mice, acetaminophen is directly toxic to LSEC via P450 activation, while in Swiss Webster mice the toxic effect on LSEC was indirectly driven by hepatocytes (DeLeve et al., 1997). APAP-induced LSEC injury precedes hepatocellular injury, supporting the hypothesis that LSECs are an early and direct target for APAP toxicity. These findings also suggest that reduced sinusoidal perfusion and increased Kupffer cell activity contribute to the development of APAP-induced liver injury (Ito et al., 2003). Although it was presented that large gaps are formed and the porosity is reduced in LSEC *in vivo*, the effects of paracetamol challenge on LSEC porosity *in vitro* have not been reported.

**Prostaglandin E1** (synthetic form: alprostadil) is a naturally occurring eicosanoid used as vasodilator for several different medical purposes (Kirtland, 1988). Applications include erectile dysfunction (ED) treatment in men who do not respond to PDE5 inhibitors (Hanchanale and Eardley, 2014) and the opening of ductus arteriosus in neonates requiring heart surgery (Singh and Mikrou, 2018). Prostaglandin E1 exerts its effect via the production of nitric oxide which stimulates soluble guanylyl cyclase to increase production of cyclic GMP (cGMP) and/or by the direct binding of prostaglandin to prostaglandin receptors, activating adenylyl cyclase to convert ATP to cyclic AMP (cAMP). The end result is the same in either pathway - decreased intracellular Ca^2+^ (Namkoong et al., 2005). Oda et al. (1997) showed that prostaglandin E1 significantly increased LSEC fenestration diameter in rat LSEC and also caused partial fusion of some fenestrations within sieve plates. They also reported increased Ca^2+^-ATPase on fenestral plasma membrane after prostaglandin E1 challenge and postulated that cytoplasmic Ca^2+^ efflux caused relaxation (and thereby dilation) of LSEC fenestrations.

**Sildenafil** (also known as Viagra) is a vasoactive agent used for the treatment of ED. It is a potent and selective inhibitor of cGMP-specific phosphodiesterase (PDE) type 5, due to its structural similarity to cGMP (Bender and Beavo, 2006). Sildenafil increases cGMP levels by inactivating PDEs that metabolize cGMP to GMP as well as by blocking ABCC5 transport protein responsible for active efflux of cGMP from the cell (Aronsen et al., 2014). cGMP is an intracellular mediator of the NO pathway that can lead to relaxion of the vascular smooth muscle (vasodilation) and thereby increase blood flow (Denninger and Marletta, 1999). Hunt et al. (2019) challenged LSEC from young (3–4 months) and old (18–25 months) mice with sildenafil and found that porosity and fenestration frequency (but not diameter) increased in LSEC from young and old mice. Sildenafil also increased cGMP levels, NO synthesis and levels of phosphorylated nitric oxide synthase (pNOS). Mao et al. (2019) also challenged LSEC (from young mice) and found that the actin rings (which delineate fenestrations) and actin stress fibers became more pronounced. In contrast to Hunt et al. (2019) and Mao et al. (2019) found that sildenafil increased fenestration diameter on average by 30%. This inconsistency might be due to the methods used – the first study used SEM to score LSEC morphology after dehydration, while the second study used dSTORM on “wet” LSEC samples. Sildenafil (and other PDE and ABC transporters inhibitors) may be an interesting therapeutic option to increase LSEC porosity in the elderly.

**Simvastatin** is a cholesterol lowering agent. Its cholesterol reducing action is via inhibition of 3-hydroxy-3-methylglutaryl (HMG) coenzyme A reductase, the rate limiting enzyme in cholesterol synthesis. Simvastatin also upregulates NO levels suggesting vascular protective effects beyond cholesterol reduction (de Sotomayor et al., 2005; Rikitake and Liao, 2005). Hide et al. (2020) reported that simvastatin was somewhat protective against warm ischemia reperfusion induced LSEC defenestration in (male Wistar) rats, so simvastatin may be able to provide a protective role in maintenance of porosity. Venkatraman and Tucker-Kellogg (2013) showed that simvastatin can antagonize Rho/ROCK (Rho-associated protein kinase) signaling, protecting from the defenestration resulting from activation of this pathway. Moreover, simvastatin treatment led to increase on both porosity and fenestration frequency in (male Wistar) rats. Interestingly these results in rats were not replicated in mice. Findings of Hunt et al. (2019) in (male C57/BL6) mice showed no significant changes in porosity or fenestration frequency in young or old mice, and only a 20% increase in mean diameter in the aged group. These findings may suggest species dependent difference in the simvastatin mechanism of action.

**TRAIL** [tumor necrosis factor (TNF)-related apoptosis-inducing ligand] is a protein ligand reported to induce cell death in transformed cells by binding to “death receptors” (Wiley et al., 1995). It is also reported to induce NO production *via* eNOS (Bartolo et al., 2015). Hunt et al. (2019) reported that LSEC challenged with lower doses of TRAIL increased LSEC porosity and fenestration frequency in young but not old mice. The lack of TRAIL response of old mice LSEC could be explained by reduced expression of TRAIL receptors in older mouse LSEC, but the level of TRAIL receptor expression in young vs. old mice remains to be determined.

### Hormones and Other Agents Acting on LSEC

#### LSEC and Vasoactive Agents

Vasoactive signaling molecules commonly act through a receptor induced relaxation in the smooth muscle surrounding the vasculature (Webb, 2003). Signaling is mostly mediated by the NO/cGMP pathway and via intracellular calcium concentrations (Chen et al., 2008). Crucially, whether a stimuli directs toward constriction or relaxation will depend on the tissue specific expression of certain receptors and the presence or absence of inhibition of parallel pathways.

Hepatic sinusoids lack smooth muscle cells but can dilate and contract responding to various vasoactive agents. Moreover, according to the two main studies addressing this issue (Oda et al., 1990; Gatmaitan et al., 1996), LSEC porosity and fenestration diameter seem to correlate with vasodilation or vasoconstriction (Table 2). These results suggest that vasodilators and vasoconstrictors have a direct effect upon the fenestrations of LSEC. The lack of super resolution techniques for living cells was one of the main drawbacks at the time of these studies of vasoactive agents’ effects on LSEC. It will be therefore beneficial for the field investigate the role of vasoconstriction and dilation in fenestration regulation using live cell imaging techniques, such as AFM, SIM or stimulated emission depletion microscopy (STED).

##### Vasodilators

**Acetylcholine** is a vasodilator acting through the cholinergic/muscarinic receptor (Sakai, 1980). In LSEC acetylcholine dilates sinusoids increasing blood flow rate and increasing fenestration diameter (Oda et al., 1990), when administered intravenously. On the other hand, **cholinergic receptor agonists** were also noted to cause narrowing of the sinusoids: **bethanechol, carbachol,** and **pilocarpine** applied topically to the liver caused constriction of the liver microvasculature, but fenestrations were not quantified (Reilly et al., 1982; McCuskey and Reilly, 1993). To further complicate these findings, intravascular admission of pilocarpine decreased while bethanechol increased the fenestration diameter. These differences in the effects can be explained by the expression of certain receptors responding to the same stimuli but having contradictory effects, however, further studies are needed. **Bethanechol** is already used as a therapy for postoperative and postpartum non-obstructive urinary retention, it would therefore be of interest to further study its effects on LSEC porosity (Oda et al., 1990). **Vasoactive intestinal peptide (VIP)** is a class II G-protein coupled receptor ligand (Umetsu et al., 2011). It has multiple physiological effects including vasodilation and increased gut motility during digestion (Iwasaki et al., 2019). VIP was shown to dilate the sinusoids and fenestra, increasing blood flow through the sinusoids which would enhance the uptake of circulating nutrients after a meal (Oda et al., 1990). **Isoprenaline** (also known as isoproterenol) is another vasodilating agent acting as a β-adrenergic receptor agonist. This G-protein is essential for cardiac function (reviewed in Wachter and Gilbert, 2012) and is used to treat bradycardia and (rarely) asthma. The effect on LSEC follows that of other of vasodilating agents increasing in both sinusoidal blood flow and fenestration diameter (Oda et al., 1990).

##### Vasoconstrictors

**Serotonin** (also known as 5-HT) is a monoamine neurotransmitter with numerous physiological functions (Berger et al., 2009). Depending on the particular receptors expressed in each vessel wall and surrounding smooth muscle tissue, serotonin can cause vasoconstriction or vasodilation in different vascular beds (Kaumann and Levy, 2006). In the liver, serotonin constricts sinusoids and reduces fenestration size (Wisse et al., 1980; Oda et al., 1990). Gatmaitan et al. (1996) showed that the effect is mediated by decreasing cAMP and increasing intracellular calcium levels in a matter of seconds. **Endothelin (ET)** is a vasoconstricting peptide that is produced in the endothelium and plays an important role in vascular homeostasis (Kawanabe and Nauli, 2011). In LSEC, it decreases both the number and the size of fenestrations (Kamegaya et al., 2002; Yokomori et al., 2006) and it reduces the blood-flow through the sinusoids (Zhang et al., 1994). Many ET receptor antagonists are used as an efficient treatment for hypertension. ET-A receptor antagonist **(BQ-123**) treatment (but not ET-B receptor antagonists) abolished ET induced defenestration and contraction of fenestrations (Yokomori et al., 2006). Blocking ET-1 activity *in vivo* by BQ-123 led to gap formation shown by SEM and TEM (Watanabe et al., 2007). The α-adrenergic receptor family mediates vasoconstriction and is coupled to guanine nucleotide regulatory proteins (G-proteins) (reviewed in Ruffolo and Hieble, 1994). **α-adrenergic receptor agonists** were found to have different effects on LSEC, **epinephrine** (adrenaline) decreased sinusoidal blood flow and contracted sinusoids and LSEC fenestrations (Oda et al., 1990), while in another study sinusoids were found slightly enlarged, and fenestrations unchanged (Wisse et al., 1980). **Norepinephrine** (noradrenaline) was found to contract sinusoids and fenestrations in both studies (Wisse et al., 1980; Oda et al., 1990). **Neuropeptide Y (NPY)**, another vasoconstrictor generally coupled to G-protein signaling, is involved in various physiological and homeostatic processes (White, 1993) but also inhibits gastrointestinal motility (Holzer et al., 2012). In LSEC, NPY constricts both sinusoid and fenestrations (Oda et al., 1990).

#### Signaling and Fenestration Maintenance

One of the most challenging aspects of studying LSEC is the dedifferentiation *in vitro* after cell extraction. LSEC lose their characteristic porous morphology after just few days in culture, significantly restricting time for experiments. There have been many attempts to slow down, stop or reverse that process (Bravo et al., 2019; Di Martino et al., 2019) but the main mechanism(s) behind the loss of fenestrations remain unknown.

**Vascular Endothelial Growth Factor (VEGF)** is a hormone that stimulates acetogenesis and angiogenesis (Apte et al., 2019). In LSEC, VEGF has been shown to increase LSEC porosity *in vitro* (Funyu et al., 2001; Yokomori et al., 2003) as well as to prolong the fenestrated phenotype of cultured LSEC *in vitro* (Xie et al., 2012b). Downregulation of VEGF signaling has been associated with LSEC defenestration, capillarization of sinusoids, and abnormal liver physiology (Carpenter et al., 2005; DeLeve, 2015). DeLeve (2015) showed that VEGF promotes fenestration formation/maintenance *via* NO-dependent and NO-independent pathways. Moreover, VEGF can induce fenestration like structures in other microvasculature, e.g., rat cremaster capillary (Roberts and Palade, 1995).

**Bone Morphogenetic Protein 9** (BMP9, also known as GDF2) is a circulating endothelial quiescence factor (David et al., 2008). In LSEC it has been indicated as necessary for fenestration maintenance and treating cells with BMP9 prolonged fenestrated phenotype in cultured LSEC (Desroches-Castan et al., 2019a). BMP9 knockouts in 129/Ola mice showed very low fenestration frequency compared to WT, without changes to diameters (Desroches-Castan et al., 2019a). However, a follow up study using C57/Black mice did not confirm these results (Desroches-Castan et al., 2019b).

**Platelet derived growth factor B (PDGF)** is a member of the PDGF family of major mitogens for many cell types (Fredriksson et al., 2004). Hepatic vascular permeability was highly increased in PDGF-B retention deficient mice, with a three-fold increase in FITC-dextran absorption and a more fenestrated phenotype (Raines et al., 2011). PDGF-B signaling is involved in pericyte recruitment and function, and stellate cell activation (Raines et al., 2011).

**Liver X receptor (LXR)** is a nuclear receptor expressed in a number of tissues, but with highest expression in the liver (Willy et al., 1995). Oxysterols are natural ligands of LXR and LXR deletion exacerbates CCl_4_ induced capillarization and basement membrane deposition (Xing et al., 2016). LXR also acts antagonistically on Hedgehog signaling (Hh) (Kim et al., 2009), while LSEC produce and respond to Hh ligands and use Hh signaling to regulate complex phenotypic changes that occur during capillarization. Moreover, inhibition of Hh using **cyclopamine** induced fenestration *in vitro* (Xie et al., 2012a).

**Plasmalemma vesicle-associated protein** (PLVAP) is associated with angiogenesis and vascular permeability, with less expression in barrier endothelium, and its expression is stimulated by VEGF (Bosma et al., 2018). PLVAP was found to be associated with a normally fenestrated phenotype, while PLVAP deficient mice present extremely low porosity and accumulation of collagen in the space of Disse (Herrnberger et al., 2014). Auvinen et al. (2019) found that there was no difference in number of fenestrations in PLVAP–/– mice, though their data shows greater variability in the knockouts. Both studies used SEM of tissue blocks for quantitative analysis of fenestrations. The difference may relate to the methods used to attain the knockouts raising the question of either knockouts being too broad/non-specific or insufficient. PLVAP mutations are associated with loss of fenestration diaphragms in other tissues (such as small intestine) (Elkadri et al., 2015).

### Lab Tools and Experimental Models

#### Experimental Animal Models for the Study of LSEC Fenestrations

Liver sinusoidal endothelial cells are the first line of defense in the liver and alterations in LSEC play a crucial role in the development of many liver diseases such as fibrosis, cirrhosis, or cancer (Gracia-Sancho et al., 2021) as well as in the age-related conditions (Hunt et al., 2018). To better understand this role, many animal models have been used. Challenge with certain drugs can mimic the development of these diseases and reduce the time and/or costs compared to waiting for them to spontaneously occur in animals (Table 3). Although the exact mechanism of action of many of these drugs is not known, the outcome is similar enough to study and propose possible treatments.

**TABLE 3:** Experimental models and lab tools affecting LSEC fenestrations.

	Fenestration diameter	Porosity	Fenestration frequency	References	Methods
**Cytoskeleton disruptors**					
Cytochalasin B	0/+	+++	+++	Braet et al., 1996a,b,c	a/b AFM, SEM, *in vitro* c SEM, TEM, *in vitro*
				Steffan et al., 1987	SEM, TEM, *in vitro* SEM, *in vivo*
				Braet et al., 1995a	TEM, *in vitro*
				Zapotoczny et al., 2017b, 2019b	AFM, *in vitro* live
				Spector et al., 1999	FL, SEM, TEM, *in vitro*
				Oda et al., 1993	SEM, TEM, *in vitro*
				Van Der Smissen et al., 1986	TEM, *in vitro*, *in vivo*
				Steffan et al., 1986	SEM, *in vivo*
				Kalle et al., 1997	AFM, *in vitro*
Cytochalasin D	0/-	+	+	Svistounov et al., 2012; Hunt et al., 2019	SEM, *in vitro*
Dihydrohalichondramide	-	nd	++	Braet et al., 2002	SEM, *in vitro*
Halihondramide	-	nd	++	Braet et al., 2002	SEM, *in vitro*
Jasplakinolide	-	nd	+	Zapotoczny et al., 2019b	AFM, *in vitro* live
				Braet et al., 1998	SEM, TEM, *in vitro*
				Spector et al., 1999	FL, *in vitro*
Latrunculin A	0	nd	++	Braet et al., 1996a	SEM, TEM, *in vitro*
				Spector et al., 1999	FL, *in vitro*
				Braet et al., 1997	SEM, *in vitro*
Misakinolide	-	nd	++	Braet et al., 1998, 1999; Spector et al., 1999	SEM, TEM, *in vitro*
Swinholide A	- -	nd	+++	Braet et al., 1998, 1999; Spector et al., 1999	SEM, TEM, *in vitro*
**Disease models**					
Dimethyl nitrosamine (DMN)	-	- -	nd	Fraser et al., 1991, 1995b; Rogers et al., 1992; Tamba-Lebbie et al., 1993	SEM, *in vivo*
Endotoxin/LPS	-/G	- -/0	-	Dobbs et al., 1994; Fraser et al., 1995b	SEM, *in vivo*
				Frenzel et al., 1977; Ito et al., 2006a	SEM, TEM, *in vivo*
				Sasaoki et al., 1995	SEM, *in vitro*
Galactosamine + endotoxin	G	nd	-	Ito et al., 2006a	SEM, TEM, *in vivo*
Galactosamine + endotoxin + matrix metaloproteinase	0	nd	0	Ito et al., 2006a	SEM, TEM, *in vivo*
Monocrotaline	G	nd	- -	DeLeve et al., 1999	SEM, TEM, *in vivo*
				DeLeve et al., 2003a, b	SEM, *in vivo*
Monocrotaline + V-PYRRO/NO	0	0	nd	DeLeve et al., 2003b	SEM, *in vivo*
Poloxamer 407	nd	nd	- -	Cogger et al., 2006	SEM, TEM, *in vitro*, *in vivo*
Pyocyanin	nd	- -	nd	Cheluvappa et al., 2007	SEM, *in vitro*
Thioacetamide (TAA)	-	- -	nd	Mori et al., 1993a,b	SEM, TEM, *in vivo*
				Xie et al., 2012b	SEM, *in vivo*
**Other**					
Superoxide anion (SOA) and nitric oxide NO	G	nd	-	Deaciuc et al., 1999	SEM, TEM, *in vivo*
7 keto cholesterol (7KC)	+	+	+	Svistounov et al., 2012; Hunt et al., 2019	SEM, *in vitro*
Antimycin A	nd	-	- - -	Zapotoczny et al., 2017b	AFM, *in vitro* live
				Braet et al., 2003	SEM, TEM, *in vitro*
Arsenic	nd	- -	nd	Straub et al., 2008	SEM, TEM, *in vitro*, *in vivo*
C3 transferase	+	+	nd	Yokomori et al., 2004	SEM, TEM, *in vitro*
Calcium ionophore	-	nd	0	Zapotoczny et al., 2019a	AFM, *in vitro*
				Oda et al., 1993	SEM, TEM, *in vivo*
Calmodulin agonist w7	+	nd	nd	Oda et al., 1993	SEM, TEM, *in vitro*
Cyclopamine	nd	+	nd	Xie et al., 2012a	SEM, *in vitro*
Diamide	nd	nd	- - -	Zapotoczny et al., 2019a	AFM, *in vitro* live
Hydrogen peroxide	+/G	- -/+	-	Cogger et al., 2001	SEM, TEM, *in vivo*
				Straub et al., 2008	SEM, TEM, *in vitro*, *in vivo*
Iodoacetic acid	nd	nd	+	Zapotoczny et al., 2019a	AFM, *in vitro* live
Lysophosphatic acid (LPA)	-	nd	- -	Yokomori et al., 2004	SEM, TEM, *in vitro*
Phorbol myristate acetate (PMA)	0	nd	-	de Zanger et al., 1997	SEM, *in vitro*
*S*-nitroso-*N*-acetyl penicillamine (SNAP)	G	nd	0	Deaciuc et al., 1999	SEM, TEM, *in vivo*
Staurosporine	0	nd	-	de Zanger et al., 1997	SEM, *in vitro*
Tert-butyl hydroperoxide	G	+	0	Cogger et al., 2004	SEM, TEM, *in vitro*, *in vivo*
Triton x100	0	- -	nd	Svistounov et al., 2012	SEM, *in vitro*
Trombospondin 1	nd	- -	- -	Venkatraman and Tucker-Kellogg, 2013	SEM, *in vitro*

*“0,” no change; G, gaps; nd, no data; increase: “+,” <50%; “++,” 50–100%; “+++,” >100%; decrease: “-,” <50%; “- -,” >50%; “- - -,” defenestration.*

Cirrhosis is a pathological liver state characterized by abnormalities in hepatic architecture such as loss of fenestrations (defenestration) and the build-up of basement membrane formed from collagen deposition in the space of Disse. Interestingly, the first stages of capillarization and defenestration was reported to be reversible prior to the deposition of collagen and formation of a basement membrane which indicates progression from fibrosis to cirrhosis (Xie et al., 2012b). Drugs such as **dimethyl nitrosamine (DMN)** or **thioacetamide (TAA**) are used to induce cirrhotic morphology in LSEC in animal models. Chronic admission of DMN (Fraser et al., 1991; Tamba-Lebbie et al., 1993) and TAA (Mori et al., 1993b; Xie et al., 2012b) was shown to lead to the loss of fenestrations, however the precise mechanism(s) behind this remains unknown. It was suggested that soluble guanine cyclase (sGC) is a crucial element of signaling necessary to maintain fenestrated LSEC morphology. sGC activation normalizes LSEC phenotype and completely prevents progression of fibrosis despite ongoing TAA exposure, so the limiting defect responsible for capillarization in this model of cirrhosis was in the NO/sGC/cGMP pathway (Xie et al., 2012b). Defenestration is an important step not only in cirrhosis and fibrosis but also with aging and its development and has an impact on the whole organism. Lack of filtration of chylomicrons and chylomicron remnants leads to hyperlipidemia (Rogers et al., 1992). Cogger et al. (2006) showed that **poloxamer 407**, a synthetic surfactant causes dramatic defenestration and massive hyperlipidemia. This finding suggests a direct role of LSEC porosity in the lipid clearance in the liver.

**Monocrotaline** has been used to a model hepatic veno-occlusive disease (DeLeve et al., 1999) and sinusoidal obstruction syndrome (SOS) (DeLeve et al., 2003a,b). Toxic effects were observed only in LSEC but not in hepatocytes nor in other parts of the endothelium. LSEC metabolize monocrotaline by conjugation to glutathione and detoxify to pyrrolic metabolite. It is believed to be a stable reproducible model resulting in a decreased number of fenestrations, gap formation and discontinuous sinusoid occurrence (DeLeve et al., 1999). It is an important reminder that LSEC also can metabolize drugs and it is not only the hepatocytes that have this function in the liver.

**Galactosamine**, together with **endotoxin** or **TNF**, causes gap formation in the sinusoids and can be used to study the neutrophil extravasation in the acute inflammatory tissue injury (Ito et al., 2006a). It was shown that inhibition of **matrix metalloproteinases**, which are involved in gap formation, reduces the neutrophil accumulation in the sinusoids. **Bacterial endotoxin** alone plays a role in the pathogenesis of cirrhosis, decreasing both number and diameter of fenestrations (Dobbs et al., 1994). Other bacterial toxins, such as **pyocyanin** or **LPS**, are used in studies of post-transplantation complications such as sepsis or ischemia-reperfusion injury. **Pyocyanin** treatment decreases porosity by its effects on the frequency of fenestrations and can be prevented by addition of catalase. This result suggests that the mechanism involves hydrogen peroxide–induced oxidative stress (Cheluvappa et al., 2007).

Another bacterial toxin, ***Clostridium botulinum* C3-like transferase (C3-transferase**), together with **lysophosphatic acid (LPA)** was tested in a study from 2004. C3-transferase is a rho inhibitor, while LPA is a rho stimulator. Rho was found to be an important regulator of the actin cytoskeleton and was therefore tested for its influence on fenestration and LSEC in general. The *in vitro* experiments on rat LSEC showed dilation and fusion of fenestrations after treatment with C3-transferase, while contraction occurred when the cells were treated with LPA. Additionally LPA caused an increase in F-actin stress fiber and actin microfilaments, while C3-transferase treatment showed the opposite (Yokomori et al., 2004).

Several models of experimental liver injury show similar morphological alterations, including gaps and ruptured sinusoids. Deaciuc et al. (1999) showed that these early changes can be mediated by the free radical species. The *in vitro* treatment of rat LSEC with **superoxide anion** or **nitric oxide** resemble the observations from *in vivo* experiments with various hepatotoxins. Treatment with **hydrogen peroxide** also increased fenestration diameter and decreased fenestration number (Cogger et al., 2001). High porosity values can be misleading in the studies where gap formation is observed so measurement of all three morphology parameters should be considered. Straub et al. (2008) presented that effect of low doses of **arsenic**, mimicking water contamination levels, also act through reactive oxygen species (ROS) generated by NADPH oxidase (NOX). This mechanism was confirmed by the protective (against arsenite) results from NOX deficient mice and use of NOX inhibitors.

#### Cytoskeleton Disruptors

Numerous agents acting on the actin cytoskeleton have significant effects on fenestration (Table 3). Two main groups include marine sponge- and mushroom-derived toxins. Relatively well-known mechanisms of action of these toxins allowed the study of the link between actin cytoskeleton and fenestrae. An extensive chapter from Braet et al. (2008), provides an overview on the *in vitro* effects of actin binding agents such as **cytochalasin B, latrunculin A, jasplakinolide A, swinholide A, misakinolide A, halichondramide, and dihydrohalichondramide**. Despite different mechanisms of promoting/inhibiting actin polymerization or fiber stabilization, all drugs result in an increase of fenestration number. The most surprising finding is the effects of jasplakinolide which promotes polymerization and stabilization of actin in other cells, but in LSEC no such effect was shown. Instead, the loss of fibers and accumulation of actin in single spots occurs within minutes of jasplakinolide treatment (Spector et al., 1999). These structures, described as ‘actin dots,’ are not fully understood, but they resemble recently described actin asters which may be connected with lipid raft reorganization (Fritzsche et al., 2017). There is an ongoing discussion about the specificity of those agents for actin. For example, cytochalasin B (but not D) was shown to influence transport of glucose across cell membranes and its overall effect can be influenced by changes in glycolysis and metabolism (Kapoor et al., 2016). **Iodoacetic acid** acts on both actin and spectrin and was shown to decrease stress filament formation. Moreover, it caused an increase in porosity and rapid opening and closing of fenestrations (Zapotoczny et al., 2019a). Nevertheless, agents acting on the actin cytoskeleton remain the most important tools for studying fenestration structure and dynamics.

#### Other Agents Affecting Fenestrations

Svistounov et al. (2012) emphasized the importance of lipid membrane stability and lipid rafts on LSEC morphology. Surfactants such as **Triton X100** or **poloxamer** showed destabilization of the cell membrane and promotion of lipid raft formation which resulted in a decrease or even complete ablation of fenestrations. Moreover, the reduction of lipid raft formation by **7 keto-cholesterol (7KC)** increased the number of fenestrations showing the connection between fenestration structure, actin and cell membrane (Hunt et al., 2019).

**Thrombospondin 1** (TSP) is a matrix glycoprotein with pro-fibrotic effects. In a study from 2013 (Venkatraman and Tucker-Kellogg, 2013) it was shown to cause dose-dependent defenestration in LSECs at 100 ng/mL. The authors additionally showed that the CD47-binding fragment of TSP1, p4N1 – which has anti-angiogenic effects in endothelial cells, also induces defenestration in LSECs.

The influence of **phorbol myristate acetate (PMA)**, a protein-kinase-C (PKC) activator and **staurosporine**, a PKC inhibitor, on LSEC have been examined by de Zanger et al. (1997). The *in vitro* treatment of rat cells for 2–7 days resulted in a decrease in porosity, due to the decrease in fenestration number without any observable change in fenestration diameter, when treated with PMA. However, despite the decrease in porosity, PMA improves LSEC cultures in terms of viability and purit, and fenestrated morphology was maintained after 7 days (de Zanger et al., 1997). Treatment with staurosporine or PMA and staurosporine showed enlarged fenestrations, gap formation and a decrease in porosity. The authors concluded that PMA acts on LSEC through PKC based on the staurosporine treatment neutralizing the PMA treatment effects.

Deaciuc et al. (1999) tested rat livers challenged with superoxide anion **[*S*-nitroso-*N*-acetyl penicillamine (SNAP)]** and nitric oxide [xanthine oxidase plus hypoxanthine (XO + HX)] generating substances. They theorized that early morphological LSEC alterations associated with liver injury are influenced by free radical species. When they perfused the rat livers with SNAP, they found a suppression of hyaluronan uptake (a test of LSEC endocytosis capacity) and the formation/creation of large gaps in LSEC morphology, sometimes instead of sieve plates, and sometimes together with fenestrations present in sieve plates.

## Mechanisms

As discussed above, a variety of agents have been tested so far showing their effect on fenestrae. Some of the agents changed the number of fenestrations, while others alter their diameters or distribution (gathered in sieve plates or individual fenestrations), including the formation of gaps. However, the clear understanding of why individual drugs have their effects on LSEC is still lacking. The main reason is that many drugs have cross-effects at the cellular level, affecting more than one cellular mechanism/pathway, including the rearrangement of cytoskeleton. Therefore, it is challenging to predict how a drug will work on LSEC fenestrations.

A thorough analysis of the effects of a variety of agents changing porosity, fenestration frequency, and fenestration diameters (including gap formation) resulted in four different hypotheses. These independent but overlapping ideas describe the possible mechanisms behind fenestration structure and dynamics.

(I) *Actin (de)polymerization regulates the number of fenestrations* (Braet et al., 1996b; Spector et al., 1999; Braet and Wisse, 2002; Mönkemöller et al., 2015). The hypothesis was discussed in Braet et al. (1995a), Braet et al. (1996b) and has been developed over the years. It was presented that the cytoskeleton plays a crucial role in the porosity of LSEC. Fenestrae-associated cytoskeleton rings (FACR) surround each fenestration and sieve plate-associated cytoskeleton surround sieve plates (Braet et al., 1995b). The application of actin (de)polymerization targeting drugs revealed the direct connection between actin cytoskeleton and fenestration number in LSEC (Spector et al., 1999; Carpenter et al., 2005). However, the disruption of actin does not destroy fenestration structure, which indicated the complex structure of FACR. Later it was reported that actin filaments surround each fenestration within a sieve plate (Mönkemöller et al., 2015).
(II) *Calcium ions regulate the diameter of fenestrations*. This second hypothesis was summed up in 2002 (Braet and Wisse, 2002). It is mainly based on the research of Oda and Yokomori presenting the role of calcium/calmodulin/actomyosin in the contractility of fenestration diameters (Oda et al., 1990; Yokomori et al., 2004). The regulation of myosin light chain (MLC) phosphorylation occurs via calcium-calmodulin signaling. Further it was suggested that MLC kinase and phosphatase may exert different effects on cell morphology (Yokomori et al., 2004).
(III) *Regulation of fenestrations depends on lipid rafts*. The sieve-raft hypothesis assumes that fenestrations are formed in the flat areas of the cell periphery, in between lipid rafts, where the cell membrane is more flexible and more prone to shape changes (Svistounov et al., 2012). Also, other ways in which lipid rafts can be connected with fenestration were proposed, such as influence on signal transduction or indirect regulation of some signaling pathways.
(IV) *Spectrin is involved in the open versus closed state of fenestration*. The hypothesis decouples the direct actin regulation from the number of fenestrations. Instead, the interplay between the membrane scaffold and actin cytoskeleton is responsible for the opening of the fenestration within the actin ring (Zapotoczny et al., 2019a).

All the above hypotheses do not exclude each other and only emphasize how complicated the mechanisms regulating the number, shape, and size of fenestrations can be. In the following subsections we will focus on the physiological regulation of number and size of fenestrations, apart from the direct (often toxic) effect of actin disturbing drugs (described above). The analysis of different agents acting on LSEC fenestrations leads to the conclusion that the phosphorylation of myosin light chain (MLC) is the core of various pathways regulating actin (de)polymerization. Calcium dependent and independent activation (phosphorylation) of MLC and release of actin binding proteins (such as tropomodulin, tropomyosin, caldesmon) leads to contraction of fenestrations and decrease in the number of fenestrations, while MLC dephosphorylation leads to the relaxation of MLC and promotes more fenestrated morphology of LSEC. The local balances regulating the levels of calcium, ROS, or NO in different parts of the cell ensure active control over the dynamics of fenestrated LSEC. The regulation covers the (de)activation of membrane proteins which may affect actin association to the membrane. Finally, the oxidative state of membrane cytoskeleton and lipid rafts distribution are additionally (passively or actively) involved in this regulation.

### Cytoskeleton

SEM and TEM allowed visualization of the fenestrae-associated cytoskeleton rings (FACR) in LSEC (Braet et al., 1996b). Preparations of “ghost” cells, after removing cell membrane with detergent, revealed a network of filaments associated with sieve plates surrounded by thicker filaments. Precise identification was not possible, but the high resolution of those techniques allowed diameter measurements suggesting a mesh of actin fibers surrounded by microtubules. The gap in the chemical information has been filled with super resolution fluorescence microscopy. Mönkemöller et al. (2015) showed the first direct correlation between the localization of cell membrane and actin around fenestration, using SIM. Recently, FACR structures could be also visualized in high resolution using AFM and dSTORM (Zapotoczny et al., 2017b, 2019a). It was also presented that the complete actin ring is necessary to form an open pore within a FACR (Zapotoczny et al., 2019a).

Cytoskeleton remodeling that influences the number of fenestrations was demonstrated for live LSEC. During the first hours after isolation LSEC spread on the substrate, opening and closing individual fenestrations and whole sieve plates. It indicated that fenestrations are not preserved from the *in vivo* to the *in vitro* state and their formation and closing is dynamic as previously suggested (Braet and Wisse, 2012). With time, the dynamics of fenestrations was shown to be slower (Zapotoczny et al., 2020). Still, fenestrations in isolated LSEC were shown to freely migrate several micrometers, and changing their diameter up to 200% during their ∼ 20 min lifespan.

Interesting labyrinth like structures have been observed *in vitro* in the proximity of the perinuclear area of LSEC (Braet et al., 2009). Some fenestrations form three dimensional multi-folded tunnels that are not always passing through the cell which contradicts the sieving role of LSEC. One possible explanation could be that these structures are caused by the cell isolation process because they have not been observed *in vivo* (in tissue samples). After digestion of the liver with Liberase/collagenase cells are detached from each other, perhaps disrupting parts of their cytoskeleton in a way that can be beyond repair after reattachment *in vitro*. Another explanation assumes that microfilament-disruption induces translocation of pre-existing three-dimensional organized fenestrae forming centers (FFCs) from the perinuclear area toward the peripheral cytoplasm (Braet et al., 1998, 2007). Recently, the formation of FFC was shown in live LSEC. It was confirmed that FFC are involved in the rapid increase in fenestration number, both in control and drug treated LSEC.

**FIGURE 3:**
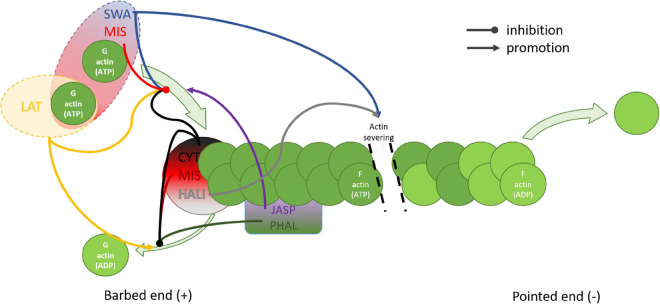
Schematic representation of the effects of actin disrupting drugs on actin filaments. Depolymerization of the barbed end of the actin filament is inhibited by CYT, MIS, and HALI which cap the barbed end, and by PHAL and JASP which attach from the side, additionally stabilizing the fiber. Latrunculin promotes depolymerization by specific sequestration of monomeric actin. Polymerization is stimulated by JASP which also binds competitively to PHAL. Barbed end polymerization is inhibited by CYT, LAT, SWA, and MIS. Both MIS and SWA bind two actin monomers, however only MIS caps the barbed end. HALI and SWA stimulate severing of the actin filament. CYT, cytochalasin; HALI, halihondramide; JASP, jasplakinolide; LAT, latrunculin; MIS, misakinolide; PHAL, phalloidin; SWA, swinholide.

The importance of the actin cytoskeleton and the structure of FACR was confirmed by the dramatic effects of any agent directly affecting actin. Actin disruptors (see Table 3 and Figure 3) were shown to rapidly induce the formation of new fenestrations (up to 300% porosity increase in 30 min by cytochalasin B) despite different mechanisms of actin depolymerization (Steffan et al., 1987; Zapotoczny et al., 2017b). Other drugs that indirectly cause actin depolymerization, such as iodoacetic acid, metformin or sildenafil, also resulted in the increase in fenestration number (Hunt et al., 2019; Zapotoczny et al., 2019a). Altogether, agents acting on actin cytoskeleton remain the most important tools in studying fenestration structure and dynamics.

Understanding the mode of action of actin disturbing agents may help us reveal fenestration structure. Actin fibers are regulated by a set of proteins such as profilin, gelsolin, or cofilin that create the dynamic, out-of-equilibrium state. Every actin-binding protein, regardless of the location of its actin-binding site, influences the adenine nucleotide exchange rate of actin and the ratio of G (monomer/globular) and F (polymerized/filamentous) actin (Figure 3). Control over that process is maintained by many signaling pathways allowing LSEC to adjust the morphology according to internal and external stimuli. Actin disrupting agents act similarly to those controlling proteins. However, they lack control or feedback loop systems therefore result in rapid and dramatic changes. The importance of the controlled signaling is especially visible in prolonged *in vitro* LSEC culture where changes in cytoskeleton, such as stress fiber formation and fenestration disappearance, occur (Yokomori et al., 2004). However, the direct relationship between the actin polymerization into the thick stress fibers and the decrease in the number of fenestrations needs to be evaluated.

In fact, actin is the only demonstrated protein that was validated to have a direct impact on the number of fenestrations. Therefore, we discuss the various signaling pathways leading to actin and actin related proteins and the ways to affect them to observe the desired effect on fenestrations in the next section.

### MLC Phosphorylation – The Core of the Fenestration’s Regulation

Myosins convert ATP to create a mechanical force on actin. Created tension in actomyosin cytoskeleton is necessary for number of cellular processes, including cell motility, cytokinesis and intracellular trafficking (Brito and Sousa, 2020). The myosins contain a neck region allowing to bind myosin light chain (MLC) domains, which are regulated by the phosphorylation and dephosphorylation via MLCK and MLCP respectively. In its phosphorylated/active form, MLC results in activation of ATP dependent myosin heavy chain binding to f-actin, which creates an active contractile force. With 30 classes of molecular motors in myosin superfamily regulating variety of cellular processes (Brito and Sousa, 2020) several reports have been dedicated to the role of MLC in the regulation of fenestration diameters. In the following subsections we focused on the cellular machinery involved in the regulation of MLC phosphorylation via calcium, NO, and ROS pathways.

### Lipid Rafts

The existence and role of lipid rafts has caused divisions in the scientific community in recent years and during The Keystone Symposium on Lipid Rafts and Cell Function (2006) the following definition was adopted: “Membrane rafts are small (10–200 nm), heterogeneous, highly dynamic, sterol- and sphingolipid-enriched domains that compartmentalize cellular processes. Small rafts can sometimes be stabilized to form larger platforms through protein-protein and protein–lipid interactions.” The role of lipid rafts in fenestrations structure and dynamics was studied only recently (Svistounov et al., 2012) and then the hypothesis of sieve-raft regulation of fenestrations was proposed by Cogger et al. (2013). Visualization with SIM revealed that rafts are not present inside sieve plates but rather surround them in an inverse distribution (Svistounov et al., 2012). Fenestrations are formed in the flat, non-raft lipid-disordered regions and are prone to changes in raft organization. 7 keto cholesterol (7KC) increases lipid ordered, non-raft regions and thus promotes fenestration formation while detergent Triton X-100 increases the relative area of raft rich regions and decreases fenestration number (Svistounov et al., 2012; Hunt et al., 2018) (causing complete defenestration at high Triton X-100 concentrations). High doses of 7KC caused gap formation and retraction of cell membrane, which can be explained by deficits in cell membranes after depletion of rafts. Another detergent, poloxamer 407, was also reported to elicit massive defenestration of LSEC (Cogger et al., 2006). Interestingly, pre-treatment with Triton X-100 (increases rafts) abrogated the effect of cytochalasin D and no increase in porosity was observed (Svistounov et al., 2012). This result elucidates the tight connection between rafts and actin cytoskeleton in fenestration structure and/or dynamics. However, it was reported that the lipid rafts in biological membranes induced by detergents may not fully resemble the normal functional rafts (Heerklotz, 2002).

Rafts are enriched in sphingolipids and cholesterol which engenders membrane stability and provides a platform for many membrane proteins that may contribute to their connection to the actin cytoskeleton (Viola and Gupta, 2007). The anchoring of actin to the lipid rafts was suggested to be realized through the FERM domain of ERM proteins and talin (Chichili and Rodgers, 2009), as well as adducin (Yang et al., 2018) and spectrin (Ciana et al., 2011). Functional rafts may not be steady-state phenomena; they might form, grow, cluster or break up, shrink, and vanish according to functional requirements, regulated by rather subtle changes in the activity (disordering or ordering) of membrane compounds (Heerklotz, 2002). These properties might be connected with the dynamic nature of fenestrations and LSEC’s ability to rapidly respond via morphology changes. The amount of lipid rafts may also have an indirect effect on fenestrations, through interactions independent of actin. It has been reported that ABC transporters, which decrease intracellular cGMP levels by its efflux, work less efficiently out of raft regions (Klappe et al., 2009). cGMP is an important signaling molecule that acts on fenestrations through PKG, decreasing intracellular calcium and promoting relaxation, both of which are connected with growing fenestration number. Lipid rafts may also affect many signal transduction pathways in the cell by serving as platforms to bring receptors into proximity with activating kinases, scaffolding proteins, and adaptor molecules that are constituent residents of lipid rafts (Rauch and Fackler, 2007).

### Spectrin

It was reported that only completely closed FACR structures contained fenestrations in the open state (Zapotoczny et al., 2019a). It was proposed that spectrin arranges actin to form a ring-like structure. Although the actin cytoskeleton is important part of fenestration structure, the membrane scaffold has a role in the regulation of opening of fenestration within FACR. In the spectrin-actin hypothesis, fenestrations can be opened if the cell height does not exceed 300–400 nm, which is double the length of the spectrin unit (Zapotoczny et al., 2019a). The proposed mechanism is based on the observation of both open and closed fenestrations within actin rings in live LSEC *in vitro*. The switch between the open and closed state was pharmacologically induced. The actin-spectrin complexes are strong enough to allow migration of the individual fenestrations across the cell membrane. Moreover, it can explain, why actin depolymerizing agents induce new fenestrations: spectrin can arrange short actin fibers to form ring like structures, and decreased cell height allows spectrin units to bind, forming new FACRs. In 2020, the role of actin/fodrin (non-erythroidal spectrin) was reported to be required in fenestration biogenesis in the endothelioma cell line bEND5, in which fenestrations can be induced pharmacologically (Ju et al., 2020). Authors showed a close association between beta actin and spectrin. Moreover, they reported that knockout of alpha spectrin resulted in 10-fold decrease in the number of fenestrations. Nevertheless, despite the increasing interest in this membrane cytoskeletal protein the knowledge of membrane skeleton regulation in endothelial cells is poorly understood.

### Regulation *via* Ca^2+^

**FIGURE 4:**
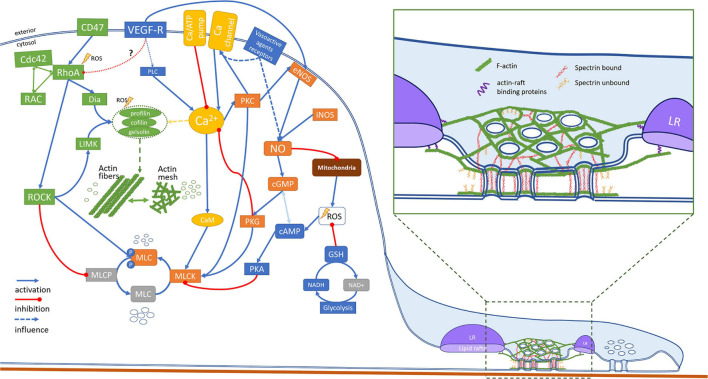
This scheme represents an attempt in unifying the proposed hypotheses of mechanisms behind the structure and dynamics of fenestrations. Various signaling pathways involved in the regulation of fenestrations in LSEC are based on the studies of LSEC (or other endothelial cells). The drugs with known mechanisms of action and reported to affect fenestrations are summarized in Table 4.

The role of calcium in the regulation of fenestration diameters was discussed by Braet and Wisse (2002). The serotonin induced influx of calcium was described to cause calcium-calmodulin dependent phosphorylation of MLCK decreasing the size of fenestrae, denoted as contraction. The reverse effect remained as speculation. Later, Yokomori et al. (2004) summarized that calcium influx affected not only MLCK, but also Rho activity. Thus, calcium can affect both MLCK and ROCK dependent phosphorylation of MLC. The authors presented results of LPA and C3 transferase, causing fenestration closing and dilating respectively, indicating that they act through MLC phosphorylation. In the Figure 4 we extended the possible regulation of MLC phosphorylation, based on the current state of knowledge. MLC is activated by the calcium mediated phosphorylation via myosin light chain kinase (MLCK) (Rigor et al., 2013). The activity of MLCK is increased by Ca^2+^-calmodulin binding and by phosphorylation by protein kinase C (PKC). PKC can also further promote MLC phosphorylation by inhibition of MLCP, however, this pathway was not confirmed in endothelium (Somlyo and Somlyo, 2000). The activation of MLCK can be hampered by the cAMP dependent kinase – protein kinase A (PKA). PKA binds to the similar region of MLCK to the Ca^2+^-calmodulin complex binding domain, hampering calcium dependent MLC phosphorylation. However, the activation of MLC is not sufficient to create a contractile force of the actomyosin complex. The actin binding proteins ensure additional control. Actin is stabilized by e.g., tropomyosin, tropomodulin, caldesmon, or calpain. The release of these proteins from actin is controlled in a calcium-concentration-dependent manner, allowing myosin to reach actin (Hepler, 2016). Moreover, the activation of actin polymerization processes, e.g., by gelsolin, profilin or cofilin is also calcium dependent and results in an increase in actin polymerization. The calcium level, regulated by calcium membrane channels and pumps or by endoplasmic reticulum release, causes a cascade of cellular mechanisms driving local changes in the cytoskeleton. These changes vary in different cells and the details of these processes is beyond the scope of this review. The contraction of actomyosin is permanent. It means that it must be actively undone to ensure actomyosin relaxation. The balance of (de)phosphorylation of MLC is maintained by MLC phosphatase (MLCP). The enzyme activity is independent of the calcium plasma concentration (Álvarez-Santos et al., 2020). In addition to the role in the dephosphorylation of MLC, it exhibits phosphatase activity toward other proteins, such as ankyrin, adducin, Tau, merlin, calcineurin-A, interleukin-16, Rb, moezin, and ezrin (Kiss et al., 2019). Inhibition of MLCP (MYPT1 complex) by activation of the RhoA/ROCK pathway, results in indirect increase in the level of phosphorylated MLC and an increase in/of the contractile forces. PKA, PKG, and PKC also cause phosphorylation of MLCP. However, a recent study showed that in contrast to the RhoA/ROCK pathway, PKG- induced phosphorylation has no effect on MLCP activity (MacDonald and Walsh, 2018). It needs to be emphasized that the phosphorylation of MLC is connected to the formation of fibrous actin (via activation of actin nucleation proteins – e.g., gelsolin, profilin, cofilin, as mentioned) and vice versa. It was suggested that actin polymerization is necessary for force development (Mehta and Gunst, 1999). Therefore, the actin relaxation/contraction state is to some extent connected with the (de)polymerization of actin. The effects of certain drugs on fenestrations may be a sum of both.

**TABLE 4:** Agents with known mechanism of action and their effects on LSEC fenestrations.

Inhibitor	Target	Effect	References
C3 transferase	RhoA	FN ↑, D ↑	Yokomori et al., 2004
Simvastatin	CD47	FN ↑, D ↑	Hunt et al., 2019
Y27635	ROCK	FN ↑	Venkatraman and Tucker-Kellogg, 2013
W7	Calmodulin	D ↑	Oda et al., 1993
7 keto cholesterol	Lipid rafts	FN ↑, D ↑	Svistounov et al., 2012
Amlodipine	Ca channel	FN ↑	Hunt et al., 2019

**Promotor/activator**	**Target**	**Effect**	**References**

LPA	RhoA	D↓	Yokomori et al., 2004
Sildenafil Amlodipine TRAIL	cGMP	FN↑	Hunt et al., 2019
Phorbol myristate	PKC	FN↓	de Zanger et al., 1997
Thrombospondin	CD47	Defenestration	Venkatraman and Tucker-Kellogg, 2013
Simvastatin	NO	FN ↑, D ↓	Venkatraman and Tucker-Kellogg, 2013; Hunt et al., 2019
Serotonin	Ca channel	D↓	Gatmaitan and Arias, 1993; Braet et al., 1995a

*FN, fenestration number; D, fenestration diameter; ↑/↓, increase/decrease.*

### Regulation *via* NO

Nitric oxide is one of the most important signaling molecules in endothelial cells and plays a crucial role in the maintenance of fenestrations in LSEC (DeLeve, 2015). NO stimulates sGC synthase and thus increases the cGMP level which then starts a cascade of signaling. cGMP stimulates the efflux of intracellular calcium into endoplasmic reticulum storage which reduces activation of MLCK through calmodulin. There are also suggestions that cGMP in microvascular endothelium can act through PKG to activate MLCP leading to further dephosphorylation of MLC (Rigor et al., 2013), but this mechanism was shown only in vascular smooth muscle cells. As described above, we propose that inactivation of MLCK together with a decrease in Ca^2+^ leads to actin relaxation, which results in the increase in fenestration diameter and/or number. There is also evidence of crosstalk between cGMP and cAMP levels which could further affect the MLC phosphorylation state (Chong et al., 2005). The exact mechanisms of action of NO on LSEC fenestration have not been described yet, however the cGMP/Ca pathway has been shown to be a part of VEGF induced NO production (Xie et al., 2012b; DeLeve, 2015). Two main sources of intracellular NO are synthases eNOS (activated among others by VEGF, endothelin, or estrogen) and iNOS (activated by cytokines during liver injuries). Both are responsible for LSEC phenotype maintenance as well as cell response to pathophysiological conditions (DeLeve et al., 2003b). The results of treatment with PMA — which activates PKC and can lead to increased NO production by eNOS — show a positive effect on maintenance of LSEC morphology *in vitro* (de Zanger et al., 1997). The effect was confirmed by co-administration of staurosporine, which inhibits PKC.

The effect of NO is complex and involves many different pathways. Besides cGMP signaling, NO can (competitively to O2) bind to complex IV in mitochondria, blocking the electron transport chain which results in an increased ROS production (Moncada and Erusalimsky, 2002). NO can then combine with ROS creating highly reactive peroxynitrate ONOO^–^. NO production by NOS is calcium dependent but at the same time NO contributes to changes in intracellular calcium. Those mechanisms seem to work as a feedback loop gently steering the cell response, especially since NO is not a stable molecule so its influence is restricted to areas local to its synthesis. In LSEC, NO is required for fenestration maintenance. However, it is not sufficient alone, and other NO independent pathways are necessary. It has been shown that, besides NO production stimulated by VEGF, NO-independent VEGF signaling is needed also (Xie et al., 2012b). We propose two possible mechanisms: in endothelial cells VEGF can act through its membrane receptor on PLC, followed release of the Ca^2+^ from the endoplasmic reticulum (Rigor et al., 2013). Then, PKC enters a feedback loop of NO production leading to a decrease in Ca^2+^. This would even further increase the NO production, but also would act as a balancing effect for calcium ions. NO can also induce protein *S*-nitrosilation, however it has been found not to affect fenestrations (Xie et al., 2012b). The other possibility is, reported in HUVEC, inhibition of Rho/ROCK pathway by VEGF receptors (Tagashira et al., 2018) which has been shown to play an important role in fenestration maintenance.

The cGMP pathway is a promising target for novel therapeutics for liver diseases and aging as restoration of cGMP levels can restore fenestrations in LSEC (Xie et al., 2012b). Drugs such as sildenafil influence cGMP by blocking its efflux by ABC transporters and degradation by phosphodiesterases (PDE) (Toque et al., 2008; Sager et al., 2012). Amlodipine, a blood pressure medication also affects fenestrations by acting through both cGMP and inhibition of Ca^2+^ channels (Berkels et al., 2004). Another drug used for lowering blood lipid levels – simvastatin, promotes NO production directly via the Akt pathway and through inhibition of Rho GTPases (de Sotomayor and Andriantsitohaina, 2001).

### Regulation *via* ROS

There are many sources of ROS within the cell, such as the mitochondrial electron transport chain, NADPH and xanthine oxidase and, highly expressed in endothelium, eNOS when uncoupled (Widlansky and Gutterman, 2011; Jerkic and Letarte, 2015). ROS were initially considered mostly as cytotoxic, but recent reports summarize their positive regulatory roles both in physiological and pathological endothelium, reviewed in Widlansky and Gutterman (2011).

Recently the cytoprotective role of ROS through activation of autophagy signaling was shown in early ischemia injury (Bhogal et al., 2018). LSEC morphology is sensitive to ROS levels and many agents act through this mechanism, such as e.g., ethanol and acetaminophen causing the disappearance of fenestrations (Deaciuc et al., 1999). *In vivo* studies showed large gaps in LSEC caused by ROS, generated by xanthine oxidase and hypoxanthine suggesting destabilization of fenestrations which also prevent cells from closing those gaps (Deaciuc et al., 1999). Glutathione (GSH) is the main physiological countermeasure to free radicals such as ROS. Reducing agents such as NAC can reduce the depletion of GSH due to the presence of oxidative stress (Sun et al., 2014). The effect of ROS on fenestrations may come from different mechanisms based on the disturbance of the redox balance in the cell. Intracellularly, mitochondria are the main source of ROS while glycolysis is the main source of reducing agents such as GSH and NADH. Scavenging of ROS directly activates the Rho/ROCK signaling pathways (Popova et al., 2010) which may lead to promotion of stress fibers. By analogy, the reduction of ROS by antioxidants should lead to reduction of Rho/ROCK signaling, therefore promoting fenestration formation. This mechanism would explain the age-related defenestration associated with higher levels of ROS and reduced redox capabilities in the cells (Herrera et al., 2010).

In endothelial cells, ROS can act as a messenger molecule activating various signaling pathways. Besides the mitochondria, a second main ROS source are NAD(P)H oxidases which can be stimulated by various vasoactive agents (Griendling et al., 2000). It has been shown that LSEC morphology is sensitive to both vasodilators and vasoconstrictors, which was shown to increase and decrease the fenestration diameter respectively (Table 2). Moreover, LSEC lack underlying smooth muscles cells to emphasize the response to vasoconstrictors/dilators. There might exist more complicated cellular mechanisms in LSEC to compensate for this. Altogether, those findings suggest that ROS may be part of signaling cascades activating redox-sensitive proteins.

## Conclusion

Drug clearance mediated by the liver is heavily dependent on the proper phenotype of LSEC, including the transport through fenestrations. Individual drugs and stimulants have been reported to influence the porosity of LSEC. Some drugs show beneficial effects on LSEC phenotype, potentially allowing re-opening fenestration (“re-fenestration”) which could be of benefit in the elderly. The role of LSEC senescence and “anti-aging” senolytic drugs, with regard to porosity, warrants further study. However, the background of polypharmacy (regular daily consumption of 4 or more medicines) in much of the elderly population needs to be considered in the refenestration context. Within this review we highlighted the areas of research which will be particularly beneficial for both physicians and researchers. LSEC research is growing in recent years and the latest stage of our knowledge about fenestrations is now facilitated with novel microscopic techniques. These super-resolution methods will continue to improve, so it is appropriate for the field to simultaneously improve sample status, for example to examine living LSEC, or “wet” fixed preparations of LSEC or whole liver mounts instead of dehydrated cells. The substrate upon which LSEC are typically cultured also likely needs to be re-worked – tissue culture plastic is considerably stiffer than the LSEC’s natural surroundings, so other softer gel-based substrates should be considered, such as those described by Guixé-Muntet et al. (2020). Ultimately, *in vivo* imaging of LSEC fenestrations *in situ* would be the ideal real-time test of refenestration therapies, but the challenges (e.g., movement due breathing and heart beat) for this type of technology are rather significant. That said, existing technologies should allow for comprehensive studies and better understanding of these unique structures, and how they work, in the coming years.

## Author Contributions

KS, LK, and CH prepared the figures and tables. PM and BZ acquired the funding. All authors took part in conceptualization, analysis and writing of the manuscript, are responsible for all aspects of the manuscript and read and agreed to the submitted version of the manuscript.

## Conflict of Interest

The authors declare that the research was conducted in the absence of any commercial or financial relationships that could be construed as a potential conflict of interest.

## Publisher’s Note

All claims expressed in this article are solely those of the authors and do not necessarily represent those of their affiliated organizations, or those of the publisher, the editors and the reviewers. Any product that may be evaluated in this article, or claim that may be made by its manufacturer, is not guaranteed or endorsed by the publisher.
